# Progressive Shifts in Oral Plaque Microbiota From Health to Coronary Artery Disease and Acute Myocardial Infarction

**DOI:** 10.14740/cr2232

**Published:** 2026-07-17

**Authors:** Han-Lin Tsai, Yung-Ping Chen, Jia-Ying Tsai, Chih-Yen Yep, Chao-Wen Tan, Chi-Pin Lee, Chun-Yu Wu, Tzu-Hsien Tsai

**Affiliations:** a Division of Cardiology, Department of Internal Medicine, Ditmanson Medical Foundation Chia-Yi Christian Hospital, Chiayi, Taiwan, Republic of China

**Keywords:** Microbiome, Myocardial infarction, Coronary artery disease

## Abstract

**Background:**

Growing evidence links oral microbial dysbiosis to atherosclerotic cardiovascular disease (ASCVD), yet its role in acute myocardial infarction (AMI) and the transition from stable coronary artery disease (CAD) to acute events remains unclear. We aimed to characterize taxonomic and functional alterations of the oral plaque microbiome across cardiovascular health states and explore their clinical relevance.

**Methods:**

We enrolled 60 age- and sex-matched adults, 20 in each group. Supragingival plaque underwent 16S rRNA sequencing and functional inference. Alpha and beta diversity were assessed, and differential features were identified by Linear Discriminant Analysis Effect Size (LEfSe). The metabolic pathway predictions were using Kyoto Encyclopedia of Genes and Genomes (KEGG) pathway.

**Results:**

Alpha and beta diversity showed no significant differences in overall microbial richness, evenness, or global community structure among groups. However, marked taxonomic shifts were observed. AMI patients exhibited enrichment of pro-inflammatory genera (*Veillonella*, *Porphyromonas*, *Dialister*, *Megasphaera*, and *Acidaminococcus*) and depletion of commensal taxa (*Haemophilus* and *Lautropia*). LEfSe identified disease-specific microbial signatures distinguishing healthy control, CAD, and AMI. Functional prediction revealed enrichment of arachidonic acid, pyrimidine, and D-glutamine/D-glutamate metabolism in CAD, with further increases in necroptosis-, proteasome-, and inflammation-related pathways in AMI, whereas two-component signaling systems were enriched in healthy controls.

**Conclusions:**

The oral microbiome exhibits progressive taxonomic and functional shifts from health to CAD and AMI, supporting an oral–cardiovascular axis and highlighting oral microbial profiles as potential noninvasive biomarkers for ASCVD risk stratification.

## Introduction

Cardiovascular disease remains a leading cause of morbidity and mortality worldwide, despite advances in primary and secondary prevention strategies [1, 2]. Accumulating evidence suggests that chronic systemic inflammation and microbial dysbiosis play critical roles in the initiation of atherosclerosis, the progression of atherosclerotic plaques, and plaque rupture leading to myocardial infarction (MI) [3, 4]. Although the gut microbiota has long been implicated in cardiovascular disease development, the oral microbiome, which harbors over 700 different types of bacterial species, is now increasingly recognized as an important contributor to cardiometabolic health [5, 6].

Epidemiological studies have consistently demonstrated that periodontal disease is associated with an increased risk of MI, independent of traditional cardiovascular risk factors [4]. Periodontopathic bacteria, including *Porphyromonas gingivalis* and *Fusobacterium nucleatum*, secrete lipopolysaccharides and virulence factors that promote systemic inflammation, endothelial dysfunction, and pro-thrombotic states [7]. Furthermore, oral–gut translocation of taxa such as *Streptococcus* spp. has been demonstrated in animal models to exacerbate myocardial injury subsequent to coronary ischemia–reperfusion [8]. Collectively, epidemiological and experimental studies underscore the oral microbiota as both a catalyst and a potential upstream driver in the pathogenesis of atherosclerosis.

Despite these advances, the composition and functional characteristics of oral microbiota from dental plaque between patients with healthy control (HC), coronary artery disease (CAD), and acute myocardial infarction (AMI) are still under-investigated. Specifically, this study seeks to characterize the oral microbiome of patients with MI and CAD in comparison with matched controls, integrate functional metagenomic analyses, and assess associations with clinical outcomes and inflammatory mediators. By delineating the oral–cardiovascular axis in AMI, we aim to identify microbial signatures that not only reflect disease severity but also provide mechanistic and therapeutic insights.

## Materials and Methods

### Study population

A total of 60 participants were enrolled between January and June 2025 at the Cardiology Department of Chiayi Christian Hospital. Subjects were assigned to three groups (n = 20 each): HCs were defined as individuals without a documented history of CAD, AMI, heart failure, stroke, peripheral artery disease, or prior coronary revascularization, matched for age (± 5 years) and sex to patient groups. Stable CAD was defined as angiographically confirmed ≥ 50% stenosis in at least one major epicardial coronary artery, clinically stable for ≥ 3 months. AMI was defined as patients presenting within 24 h of symptom onset and meeting the definition of MI according to guidelines [9].

### Inclusion and exclusion criteria

All participants were aged 40–80 years and provided written informed consent. Exclusion criteria included antibiotic or probiotic use within 8 weeks; active oral infection aside from chronic periodontitis; chronic inflammatory or autoimmune diseases; malignancy; or recent (< 3 months) acute infection.

### Oral plaque collection

Prior to any dental cleaning or antimicrobial mouthwash, supragingival plaque was collected from the buccal surfaces of the first molars using sterile Gracey curettes. Plaque material was immediately placed into 1.5 mL DNA/RNA Shield solution (Zymo Research) and stored at –80 °C until processing.

### DNA extraction and 16S rRNA gene sequencing

Paired-end reads were quality-filtered with a Q20 cutoff and merged; non-merged reads and merged fragments shorter than 400 bp were excluded. The remaining sequences were taxonomically classified by BLAST alignment against the NCBI microbial 16S rRNA database using CLC Genomics Workbench v8.5, retaining only hits with ≥ 96% sequence identity for downstream analyses. Only reads exhibiting ≥ 97% sequence identity to their top BLAST hit were retained for downstream analysis.

### Bioinformatic analysis

Raw sequencing reads were processed with Trimmomatic v0.39 to remove adapter sequences and trim low-quality bases (Phred score < 20). Denoising and amplicon sequence variant (ASV) inference were performed in QIIME 2 v2024.2 using DADA2, yielding an ASV abundance table for downstream analyses. Within-sample diversity was assessed by calculating abundance-based coverage estimator (ACE), Chao1, Shannon, Simpson, and observed richness indices in QIIME 2, providing metrics of microbial richness and evenness. Between-sample community dissimilarity was evaluated using Bray–Curtis distance; principal coordinate analysis (PCoA) was conducted and visualized in R (vegan package) to depict sample clustering patterns. ASVs were taxonomically annotated to phylum and genus levels against the SILVA 138 reference database; relative abundances of dominant taxa were plotted as stacked bar charts using the ggplot2 package in R. For the experimental cohort, differential biomarkers were identified via a Kruskal–Wallis test followed by pairwise Wilcoxon rank-sum tests and Linear Discriminant Analysis Effect Size (LEfSe) to pinpoint taxa with significant intergroup differences. Functional profiles were inferred from the ASV table using Tax4Fun2; pathway abundances were compared across groups in STAMP, with visualization of significantly altered Kyoto Encyclopedia of Genes and Genomes (KEGG) modules.

### Statistical analysis

Continuous variables are presented as mean ± standard deviation (SD) or median with interquartile range (IQR), as appropriate. Differences among the three groups were assessed using one-way analysis of variance (ANOVA) for normally distributed variables or the Kruskal–Wallis test for non-normally distributed variables. When overall group differences were significant, *post hoc* pairwise comparisons were performed using Wilcoxon rank-sum tests. To account for multiple testing and reduce the risk of false-positive findings, P values from pairwise comparisons, differential taxa analyses, and predicted pathway analyses were adjusted using the Benjamini–Hochberg false discovery rate method where applicable. Categorical variables were compared using the χ^2^ test or Fisher’s exact test, as appropriate. All statistical analyses were performed using R version 4.2.2. A two-sided P value or false discovery rate–adjusted P value < 0.05 was considered statistically significant.

### Ethical statements

The study was conducted in accordance with the Declaration of Helsinki and was approved by the Institutional Review Board of Ditmanson Medical Foundation Chia-Yi Christian Hospital, Chiayi, Taiwan (IRB No. IRB2023050).

## Results

### The diversity of oral microbiota in patients with AMI compared with CAD and HCs

There were 20 HCs, 20 patients with CAD, and 20 patients with AMI included in this study. The baseline characteristics of HCs and patients with CAD and MI are presented in Table 1. No significant differences were observed in baseline demographic and clinical characteristics among the three groups. However, the use of statins is less in the HC group than in the CAD and AMI groups.

**Table 1:** Baseline Demographic and Clinical Characteristics of the Study Population

	Healthy control (N = 20)	CAD (N = 20)	MI (N = 20)	P value
Age	59 ± 17.2	60 ± 11.4	61 ± 16.4	0.874
Male	11 (73.3%)	12 (80%)	16 (80%)	0.874
HTN	3 (15%)	4 (20%)	6 (30%)	0.798
DM	1 (5%)	2 (10%)	4 (20%)	0.529
Smoke	1 (5%)	3 (15%)	7 (35%)	0.131
Hyperlipidemia	2 (10%)	3 (15%)	5 (25%)	0.649
Periodontal disease (mild/moderate/sever)	13/6/1	8/9/3	7/8/5	
Statin treatment	2 (10%)	13 (65%)	3 (15%)	< 0.001
Antiplatelet drugs	0 (0%)	20 (100%)	20 (100%)	< 0.001

CAD: coronary artery disease; DM: diabetes mellitus; HTN: hypertension; MI: myocardial infarction.

**Figure 1:**
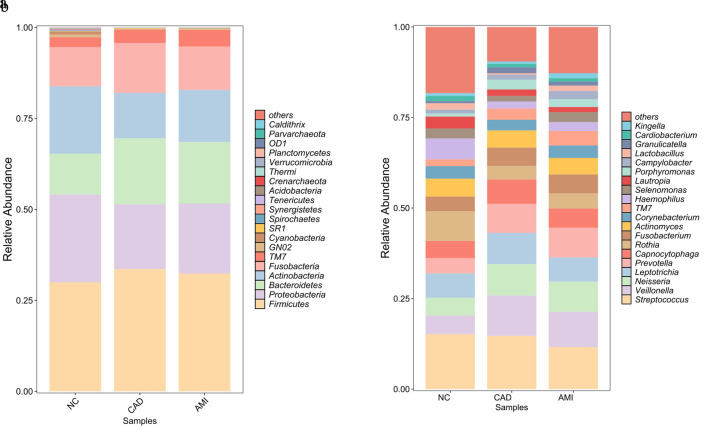
Taxonomic composition of the oral plaque microbiome across study groups. (a) Relative abundance of dominant bacterial phyla in oral plaque samples from HCs, patients with CAD, and patients with AMI. Firmicutes, Bacteroidetes, Proteobacteria, Fusobacteria, and Actinobacteria were the predominant phyla across all groups, with a higher abundance of Bacteroidetes and TM7 and reduced Proteobacteria observed in the AMI group. (b) Relative abundance of key bacterial genera among the three groups. Patients with AMI exhibited enrichment of genera including *Veillonella*, TM7, and *Neisseria*, whereas commensal genera such as *Haemophilus* and *Lautropia* were reduced. AMI: acute myocardial infarction; CAD: coronary artery disease; HC: healthy control.

At the phylum level, Firmicutes, Bacteroidetes, Proteobacteria, Fusobacteria, and Actinobacteria were the most common taxa in all of the samples. However, the relative abundance of these phyla differed markedly among the groups. Figure 1a illustrates the relative distribution of the predominant bacterial phyla. Compared with the HC group, the AMI group exhibited a significantly higher relative abundance of Bacteroidetes and TM7, accompanied by a markedly lower abundance of Proteobacteria (Fig. 1a). At the genus level, distinct microbial signatures were observed among the three groups. The AMI group demonstrated a significantly increased relative abundance of genera such as *Veillonella*, TM7, and *Neisseria*. In contrast, commensal and health-associated genera, including *Haemophilus* and *Lautropia*, were significantly reduced in patients with AMI (Fig. 1b). Collectively, these results indicate a pronounced shift toward an oral microbial dysbiosis in patients with AMI, characterized by an enrichment of opportunistic taxa and a depletion of beneficial commensal bacteria.

### Alpha and beta diversity analysis

**Figure 2:**
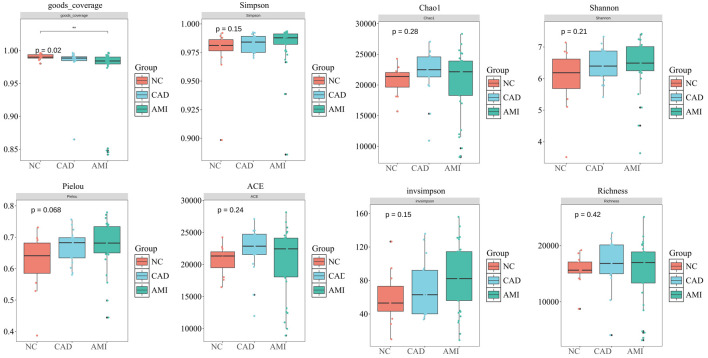
Alpha diversity of the oral plaque microbiome. Alpha diversity indices including Shannon, ACE, Chao1, observed richness, inverse Simpson, Simpson, and Pielou’s evenness were used to evaluate within-sample microbial diversity in HC, CAD, and AMI groups. No significant differences were observed among the three groups (all P > 0.05), indicating similar microbial richness and evenness. ACE: abundance-based coverage estimator; AMI: acute myocardial infarction; CAD: coronary artery disease; HC: healthy control.

**Figure 3:**
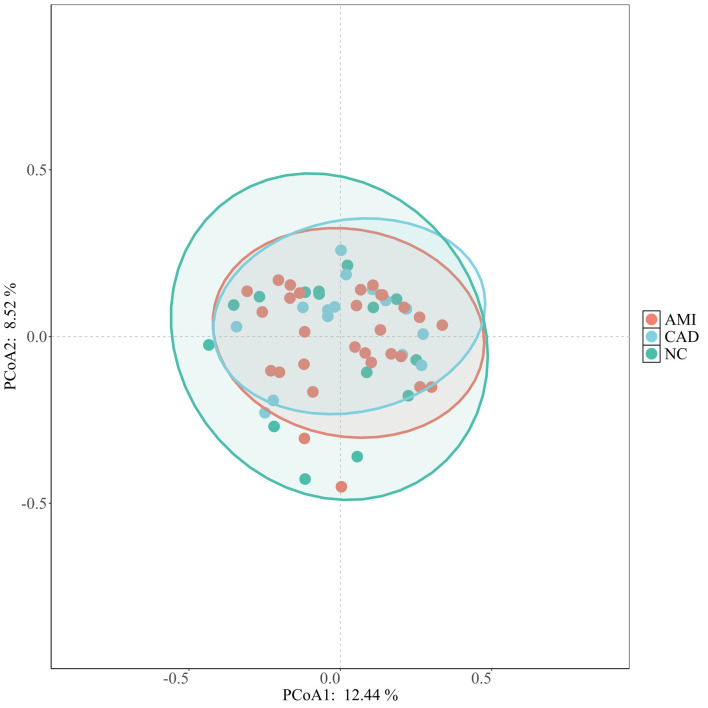
Beta diversity analysis of oral microbiome composition. Principal coordinate analysis (PCoA) based on Bray–Curtis dissimilarity illustrating the overall microbial community structure among HC, CAD, and AMI groups. No clear clustering by disease status was observed, suggesting that the global microbial community composition remained broadly similar among the groups. AMI: acute myocardial infarction; CAD: coronary artery disease; HC: healthy control.

We further assessed within-sample (alpha) diversity of the oral plaque microbiome across the HC, CAD, and AMI groups by using seven complementary diversity indices (Fig. 2). We measured the alpha diversity of the oral microbiome from plaque using the Shannon index, ACE estimator, Chao1, observed richness, inverse Simpson, Simpson, and Pielou in indexes between three groups. There were no statistically significant differences in alpha diversity among the HC, CAD, and AMI groups across all indices assessed (all P > 0.05), indicating similar microbial richness and evenness within samples. We then examined between beta diversity to assess overall microbial community structure among the groups. Principal coordinate analysis (PCoA) utilizing Bray–Curtis dissimilarity did not demonstrate significant clustering by disease status, suggesting that the overall microbial community composition was not markedly different among the three groups (Fig. 3). These results indicate that although taxonomic shifts transpire (as illustrated in Fig. 1), global diversity metrics persist unchanged across health and disease states, and overall microbial diversity and community structure remain largely preserved across health and disease states.

### Differential taxa analysis by LEfSe

**Figure 4:**
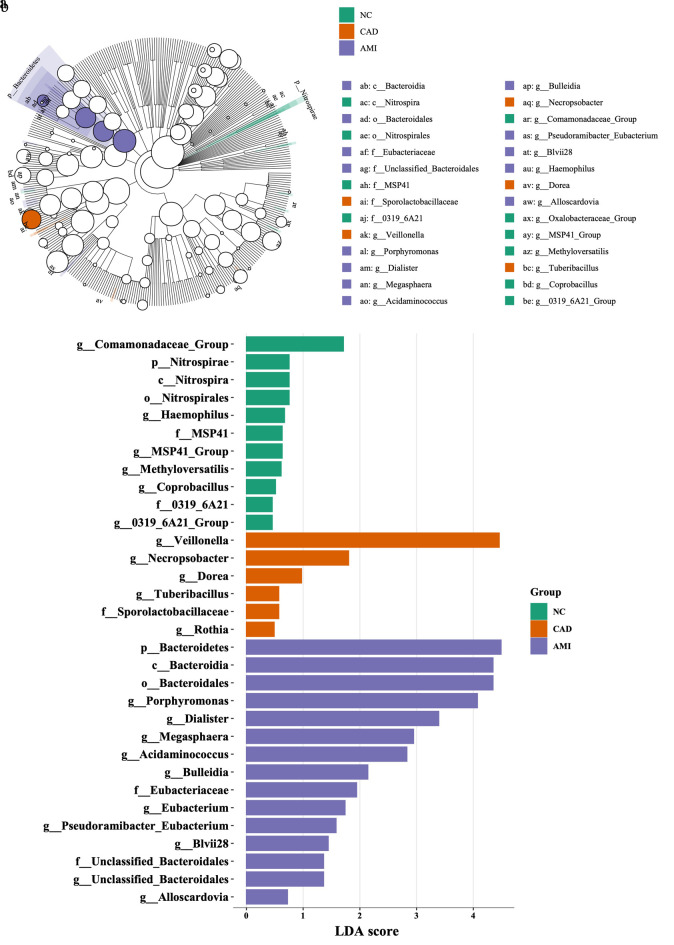
LEfSe analysis identifying disease-specific microbial taxa. (a) Cladogram showing differentially enriched taxa across HC, CAD, and AMI groups identified by LEfSe. Taxa with logarithmic LDA scores > 3.0 and P < 0.05 were considered significant. Green, orange, and purple branches represent taxa enriched in HC, CAD, and AMI, respectively. Uncolored nodes indicate taxa without significant differences. (b) Histogram of LDA scores for taxa differentially abundant among the three groups, highlighting microbial signatures associated with each cardiovascular state. AMI: acute myocardial infarction; CAD: coronary artery disease; HC: healthy control; LDA: linear discriminant analysis; LEfSe: Linear Discriminant Analysis Effect Size.

To identify differentially enriched taxa across groups, we performed LEfSe based on 16S rRNA gene taxonomic profiles from the three groups—HCs, patients with CAD, and patients with AMI. Taxa with a logarithmic linear discriminant analysis (LDA) score > 3.0 and P < 0.05 were considered significantly discriminative and are presented in Figure 4, with the cladogram shown in the upper panel and the corresponding LDA scores displayed as horizontal bar plots in the lower panel. The circular cladogram highlights phylogenetic lineages that are differentially enriched among the three groups. Branches colored in green represent taxa overrepresented in HCs, orange indicates taxa enriched in CAD, and purple denotes taxa enriched in AMI (Fig. 4a). Uncolored nodes correspond to taxa that did not reach statistical significance. In the HC group (Fig. 4b, green bars), 10 genera and higher-order taxonomic clades were significantly enriched. Several of these taxa represent common oral commensals, including *Haemophilus*, *Comamonadaceae* group, *Methyloversatilis*, and *Coprobacillus*. The LDA scores of these taxa ranged from approximately 0.5 to 1.8, consistent with a microbiome profile associated with oral health.

In the CAD group (orange bars), five taxa were significantly enriched compared with the other groups, indicating a modest but discernible shift in oral microbial composition. The most prominently enriched taxon was *Veillonella*, with an LDA score of approximately 4.2, followed by *Dorea*, *Tuberibacillus*, *Sporolactobacillaceae*, *Rothia*, and *Dora*. These alterations suggest an early deviation from a healthy oral microbial community in patients with stable CAD.

The AMI group exhibited the most pronounced microbial dysbiosis, with 15 significantly enriched lineages (purple bars). These taxa included anaerobic and facultative anaerobic genera, such as *Porphyromonas*, *Dialister*, *Megasphaera*, *Acidaminococcus*, *Bulleidia*, *Eubacterium*, and *Alloscardovia*. The LDA scores for these taxa ranged from approximately 0.7 to 4.2, indicating marked overrepresentation of potentially pathogenic or inflammation-associated bacteria in the context of acute myocardial injury.

### Functional pathway analysis

**Figure 5:**
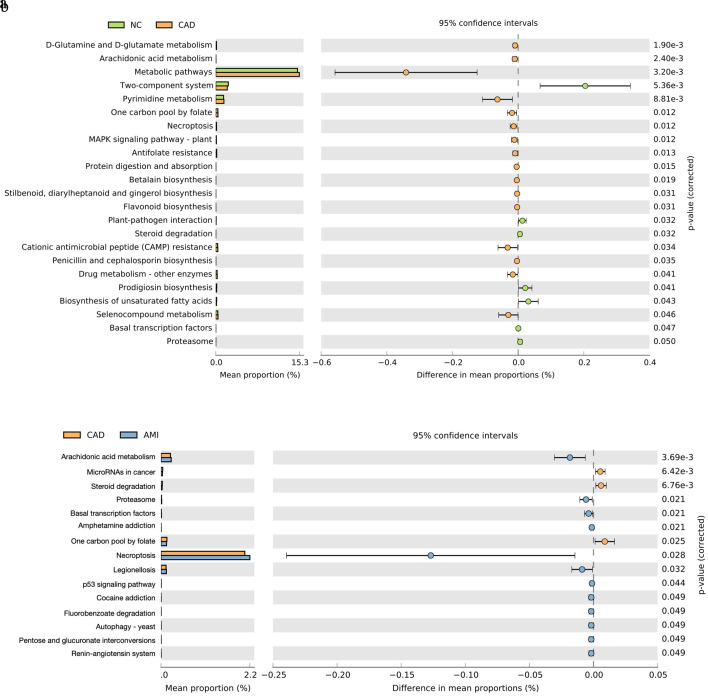
Predicted functional pathways of the oral microbiome. (a) Differential KEGG pathway enrichment between HC and CAD groups predicted using Tax4Fun2. CAD samples showed increased pathways related to arachidonic acid metabolism, pyrimidine metabolism, and D-glutamine/D-glutamate metabolism. (b) Comparison of predicted pathways between CAD and AMI groups. AMI samples exhibited further enrichment of pathways associated with necroptosis, proteasome activity, and inflammation-related processes, while two-component signaling systems were more abundant in healthy controls. AMI: acute myocardial infarction; CAD: coronary artery disease; HC: healthy control; KEGG: Kyoto Encyclopedia of Genes and Genomes.

To investigate the potential functional implications of the observed taxonomic alterations, we performed predictive functional profiling of the oral microbiome using KEGG orthologs inferred by Tax4Fun2. Compared with HCs, the CAD group exhibited significant upregulation of pathways related to D-glutamine and D-glutamate metabolism, arachidonic acid metabolism, pyrimidine metabolism, and global metabolic activity (Fig. 5a). In contrast, two-component signaling systems were more prominently enriched in HCs, reflecting preserved microbial homeostatic signaling capacity (Fig. 5b).

Direct comparison between the CAD and AMI groups revealed that arachidonic acid metabolism was further upregulated in patients with AMI. In addition, pathways associated with necroptosis, proteasome function, and legionellosis were significantly enriched in the AMI group compared with the CAD group, suggesting an enhanced inflammatory and stress-response signature during acute myocardial injury.

In summary, Tax4Fun2-based functional inference indicates that the transition from health to stable CAD is characterized by a marked shift toward pro-inflammatory and proliferative metabolic pathways, accompanied by a reduction in microbial signaling pathways associated with homeostasis. These functional perturbations are largely maintained during progression from CAD to AMI, with selective amplification of inflammatory and cell-death–related pathways rather than extensive global reprogramming of the microbial metagenome.

## Discussion

A growing body of evidence supports causal relationship between oral microbiota and atherosclerotic cardiovascular disease (ASCVD). Chronic periodontal inflammation and translocated oral pathogens are linked to endothelial dysfunction, plaque formation, and systemic immune activation [3, 4, 10, 11], which are the major mechanisms that lead to ASCVD. Oral microbial communities were recently characterized in patients with chronic CAD [12–14], but the microbiome signature of oral plaque associated with patients with AMI remains largely unexplored.

Most prior studies have primarily focused on taxonomic composition or periodontal disease severity, often overlooking functional inference and the dynamic transition from stable CAD to acute coronary syndromes. In addition, relatively few investigations have systematically compared both compositional alterations and taxonomic shifts across disease stages. Moreover, functional metabolic reprogramming within the oral microbial community—particularly pathways that may contribute to plaque instability and thrombotic events—has been rarely examined [7]. Given the accessibility of the oral cavity and its role as a portal to the systemic circulation, a deeper understanding of oral microbial dysbiosis and its metabolically active potential during acute cardiac events may provide novel insights into cardiovascular disease pathogenesis and offer opportunities for noninvasive risk stratification and biomarker development [15, 16]. Accordingly, to identify taxa and metabolic pathways associated with acute myocardial injury, we sought to elucidate the role of the oral microbiota in modulating cardiovascular events and to explore its potential utility as an early indicator of disease severity.

### Oral microbiota alterations in cardiovascular disease

First, the study found that patients with AMI, stable CAD, and HC had different taxonomic and functional alterations in their oral plaque microbiota. These results may have implications for disease progression, risk assessment, and microbiome-targeted therapeutic strategies, and further support accumulating evidence linking the oral microbiota to systemic inflammation and cardiovascular disease [12–14].

Patients with AMI had higher relative abundances of genera like *Veillonella*, TM7, and *Neisseria* at the compositional level. The previous reports have been linked the abundance of *Veillonella*, TM7, and *Neisseria* taxa with oral dysbiosis and inflammation conditions [17, 18]. Notably, *Veillonella* particularly has been contributed to lactic acid fermentation and nitrate metabolism, generating an acidic environment that promotes microbial imbalance and inflammatory process [17]. On the other hand, commensal genera such as *Haemophilus* and *Lautropia*, which are known for maintaining oral immune homeostasis, were markedly reduced in AMI, indicating a loss of microbial symbiosis. Emerging evidence suggests that species within the *Haemophilus* genus interact with mucosal immune barriers to maintain epithelial integrity and suppress pro-inflammatory signaling pathways [19]. This equilibrium seems to be upset in AMI patients, who also have lower *Haemophilus* abundance. These results suggested that *Haemophilus* might be used as a probiotic, but more investigation is required to validate this theory.

Collectively, these findings raise the possibility that beneficial oral commensals, particularly *Haemophilus*, may play a protective role in cardiovascular health. While speculative, this observation supports the concept that restoration of specific oral microbial taxa could represent a novel probiotic or microbiome-modulating strategy. Further mechanistic and interventional studies are warranted to validate this hypothesis and to elucidate the causal role of oral commensals in cardiovascular disease pathogenesis.

### LEfSe-derived disease-specific taxa

Although overall richness and evenness of the oral microbiome were largely preserved across cardiovascular health states, as reflected by the absence of significant differences in conventional alpha and beta diversity metrics, LEfSe analysis revealed distinct disease-specific microbial signatures. These findings underscore that microbial community composition, rather than global diversity indices alone, may play a more critical role in the pathogenesis of CAD and AMI.

In the HC group, LEfSe identified nine significantly enriched taxa, including *Haemophilus*, *Comamonadaceae* group, *Methyloversatilis*, and *Coprobacillus*, with LDA scores ranging from approximately 0.5 to 1.8. These taxa are generally regarded as beneficial commensals involved in mucosal immune regulation and inflammation control. Notably, nontypeable *Haemophilus influenzae* has been shown to induce Foxp3^+^ regulatory T cells, thereby attenuating inflammatory responses [19, 20]. Enrichment of *Comamonadaceae* in zebrafish correlates with reduced IL-1β expression and lower pro-inflammatory cytokines in a previous study [21]. Similarly, *Coprobacillus cateniformis* (*C. cateniformis*) is an immunomodulatory bacterium capable of shaping T-cell–mediated immunity, with emerging evidence supporting its potential therapeutic relevance in cancer immunotherapy [22].

In contrast, the AMI cohort exhibited pronounced oral microbial dysbiosis, characterized by the significant enrichment of 15 predominantly anaerobic lineages, consistent with an overrepresentation of classical periodontal pathogens and pro-inflammatory taxa. Among these, *Porphyromonas gingivalis* is known to secrete gingipains and lipopolysaccharides that disrupt epithelial barriers, promote bacteremia, and activate endothelial Toll-like receptor (TLR)-2 and TLR-4 signaling, thereby contributing to vascular inflammation and atherogenesis [23]. *Dialister pneumosintes* produced other metabolites that activate the NLRP3 inflammasome, leading to the upregulation of IL-1β, IL-6, TNF-α, MMP-2, and MMP-9, which are associated with fibrous cap degradation and plaque rupture [24]. In addition, amino-acid–fermenting genera, including *Megasphaera* and *Acidaminococcus*, may further exacerbate vascular inflammation through local acidification and oxidative stress, thereby creating a microenvironment conducive to plaque instability [25]. Taken together, the enrichment of these pro-inflammatory and metabolically active taxa at the onset of AMI highlights their potential utility as noninvasive microbial biomarkers of plaque vulnerability, complementing established clinical markers such as cardiac troponin and C-reactive protein. These findings further support a contributory role of oral microbial dysbiosis in the transition from stable atherosclerosis to acute coronary events.

### Functional signatures of oral microbiome in CAD and AMI

Based on predicted functional profiling, the CAD group showed enrichment of pathways related to pyrimidine metabolism, arachidonic acid metabolism, and D-glutamine/D-glutamate metabolism. These findings suggest an increase in microbial capacity for nucleotide biosynthesis, amino-acid turnover, and production of pro-inflammatory lipid mediators, which may contribute to plaque destabilization and thrombogenesis [26–29].

These pathways were further amplified in patients with AMI, who exhibited significantly increased necroptosis, proteasome, and Legionellosis-associated functions. Collectively, these pathways reflect enhanced microbial stress adaptation, virulence-associated signaling, and host cell death–related responses, which may exacerbate endothelial injury and impair post-infarction recovery [28, 30].

In contrast, two-component signaling systems—key regulators of environmental sensing, quorum signaling, and microbial community stability—were most enriched in HCs and markedly reduced in disease states. This pattern suggests loss of microbial homeostatic signaling and ecological disruption of the oral niche, with a shift toward pathobiont dominance [30]. Taken together, these findings support the concept of an “oral–cardiovascular axis,” previously described in gut dysbiosis studies [6, 8], and extend this paradigm to the oral microbiome. Recent evidence has increasingly supported a broader relationship between microbiome dysbiosis and cardiovascular disease [31]. In a recent systematic review and meta-analysis, Albulushi and Taha summarized updated evidence linking gut microbiome dysbiosis with heart failure severity and outcomes, highlighting potential mechanisms involving impaired barrier function, systemic inflammation, microbial metabolites such as trimethylamine-N-oxide and short-chain fatty acids, modulating immune responses and improving myocardial energy metabolism. Although the present study focused on the oral rather than gut microbiome, these findings support the concept that dysbiosis at mucosal microbial sites may reflect systemic cardiovascular vulnerability. In this context, the oral microbiome may serve not only as a marker of local oral health but also as a potential noninvasive indicator of broader inflammatory and metabolic perturbations associated with CAD and AMI. Nevertheless, whether oral microbial alterations directly contribute to coronary plaque instability or myocardial injury remains to be determined in future longitudinal and mechanistic studies. Our results indicate that oral microbial communities may actively contribute to cardiovascular pathology through coordinated metabolic and inflammatory signaling.

Beyond conventional cardiovascular risk assessment, innovative imaging modalities may help clarify the clinical significance of oral microbiome dysbiosis. Speckle tracking echocardiography (STE), particularly global longitudinal strain assessment, can detect subclinical myocardial dysfunction even when left ventricular ejection fraction remains preserved. Recent evidence in cardiometabolic disorders suggests that myocardial deformation imaging may reveal subtle impairment in left ventricular mechanics before overt cardiac disease becomes clinically apparent [32]. Therefore, integrating oral microbiome profiling with STE-derived myocardial strain parameters may represent a promising multimodal strategy for earlier cardiovascular risk stratification. Oral microbial dysbiosis may be linked to systemic inflammation, myocardial inflammation, and adverse myocardial remodeling, which could potentially be reflected by impaired strain parameters. Future prospective studies are warranted to evaluate the relationship between oral microbiota composition, inflammatory or microbial metabolites, and myocardial strain indices, thereby providing further mechanistic insight into the oral–cardiovascular axis.

### Limitations

Several limitations of this study should be acknowledged. First, the cross-sectional design precludes determination of temporal or causal relationships between oral microbial dysbiosis and acute myocardial events; therefore, it remains unclear whether the observed microbial shifts precede or follow plaque destabilization. Second, the relatively small sample size and single-center recruitment may limit generalizability, reduce statistical power to detect weaker associations, and leave residual confounding from factors such as medication use, dietary habits, and oral hygiene practices. Third, 16S rRNA gene sequencing provides taxonomic profiling primarily at the genus level, limiting the ability to distinguish strain-specific virulence traits. Future studies using shotgun metagenomics or culture-based approaches are needed to differentiate commensal from pathogenic lineages with higher resolution. Fourth, the KEGG pathway findings in this study were inferred using Tax4Fun2 based on 16S rRNA gene sequencing data. Therefore, these results should be interpreted as predicted functional potential rather than direct evidence of microbial gene content or metabolic activity. Such inference cannot substitute for shotgun metagenomic sequencing, metatranscriptomic analysis, metabolomic profiling, or experimental validation. Therefore, future studies integrating multi-omics approaches are required to confirm whether the predicted pathways identified in this study are functionally active and biologically relevant in CAD and AMI. Finally, supragingival plaque samples were collected using a standardized approach; detailed periodontal parameters, including probing depth, clinical attachment loss, bleeding on probing, dentition status, recent dental procedures, and oral hygiene practices, were not systematically assessed. In addition, dietary habits and certain medication exposures, including statins, antiplatelet drugs, may influence oral microbial composition. Therefore, residual confounding related to oral health status and lifestyle factors cannot be excluded. In particular, statins, antiplatelet agents, may alter microbial communities or systemic inflammatory status. Due to the relatively small sample size, we were unable to perform robust medication-stratified or fully adjusted microbiome analyses. Therefore, residual confounding related to CAD therapy cannot be excluded.

### Conclusions

The oral microbiome exhibits progressive taxonomic and functional alterations from health to stable CAD and AMI, characterized by increasing pro-inflammatory and pathogenic signatures in this single-center cross-sectional study. These findings suggest an association between oral microbiome dysbiosis and coronary disease status but should be interpreted as exploratory and hypothesis-generating. Larger longitudinal studies integrating periodontal assessment, shotgun metagenomics, metabolomics, and host inflammatory profiling are warranted to validate these observations and clarify potential mechanistic links.

## Data Availability

The 16S rRNA sequencing data generated in this study have been submitted to a public repository under submission ID SUB16093861. The accession number will be provided once available. Other data supporting the findings of this study are available from the corresponding author upon reasonable request.

## Abbreviations

ACE: angiotensin-converting enzyme
AMI: acute myocardial infarction
ASCVD: atherosclerotic cardiovascular disease
CAD: coronary artery disease
HC: healthy control
KEGG: Kyoto Encyclopedia of Genes and Genomes
LDA: linear discriminant analysis
LEfSe: Linear Discriminant Analysis Effect Size
NCBI: National Center for Biotechnology Information
PCoA: principal coordinate analysis
QIIME 2: Quantitative Insights Into Microbial Ecology 2
STAMP: Statistical Analysis of Metagenomic Profiles
STE: speckle tracking echocardiography
